# Caring amid crisis: a qualitative study of caregivers' experiences, wellbeing, and support for older adults

**DOI:** 10.3389/fpubh.2026.1848846

**Published:** 2026-07-03

**Authors:** Sheila A. Boamah, Kyla J. Kovalik

**Affiliations:** 1 School of Nursing, Faculty of Health Sciences, McMaster University, Hamilton, ON, Canada; 2 Department of Health, Aging & Society, Faculty of Social Sciences, McMaster University, Hamilton, ON, Canada

**Keywords:** care coordination, caregiver experiences, health and social care systems, older adults, Ontario, quality of life, unpaid caregivers

## Abstract

**Background:**

Unpaid caregivers are a critical yet structurally under-recognized component of health and social care systems, providing essential support to older adults living with chronic illness, frailty, and complex care needs. Although caregiving challenges predate COVID-19, the pandemic intensified reliance on unpaid care and exposed persistent gaps in system coordination, service access, and caregiver support. This study examines how unpaid caregivers in Ontario, Canada, retrospectively understand caregiving during and following this period, with attention to how health and social care system structures shape caregiver recognition, access to supports, and experiences of strain.

**Methods:**

This qualitative study used an interpretive description design and drew on semi-structured interviews with caregivers conducted between November 2024 and September 2025. Interviews were completed in person, by telephone, or via Zoom and analyzed using reflexive thematic analysis within an iterative interpretive framework. Analysis was guided by an interest in both experiential accounts of caregiving and the institutional arrangements through which care is organized, including caregiver identification practices, assessment processes, service coordination, and access to formal supports. Coding and theme development were iterative and comparative, occurring alongside data collection to allow emerging insights to inform subsequent interviews. Analytic rigor was supported through sustained team reflexivity, iterative coding cycles, and regular analytic discussions to refine interpretations and ensure coherence across themes.

**Results:**

Fifteen caregivers participated. Analysis generated four interconnected themes: (1) establishing a caregiving identity; (2) experiencing emotional strain and unmet support needs; (3) maintaining social connection through diverse support networks; and (4) managing daily caregiving demands and accessing respite. Across themes, caregiving was shaped by interactions with health and social care systems that influenced whether caregivers were recognized, supported, and connected to appropriate resources. Participants described persistent challenges in navigating services, accessing emotional and practical supports, sustaining social connections, and obtaining respite.

**Conclusions:**

Caregiver strain arises not only from the demands of caregiving, but also from how caregiving is organized and operationalized within health and social care systems. By identifying gaps in caregiver recognition, assessment, coordination, and support, this study shifts attention from individual burden to the institutional conditions shaping caregiver experiences. Strengthening caregiver support will require integrated system responses that embed caregivers within routine care pathways and position them as essential partners in care delivery.

## 1 Introduction

Unpaid caregivers are a foundational yet structurally under-recognized component of contemporary health and social care systems. As populations age and the prevalence of multimorbidity and long-term care needs increases, health systems have become increasingly reliant on family and friend caregivers to sustain care across home, community, and institutional settings. In Canada, this reliance is substantial: approximately 13.4 million individuals were engaged in unpaid caregiving in 2024, and roughly one in four adults aged 15 years and older has provided care to someone with ongoing health-related needs (1, 2). These figures reflect not only the ubiquity of caregiving as a social role, but also its embeddedness within the functioning and sustainability of health systems.

Despite this structural dependence, caregiving is not consistently integrated into the design, delivery, or evaluation of health and social care services. Care is frequently organized around patients rather than care networks, resulting in systems that rely on unpaid caregivers while only partially recognizing their roles within care processes (3, 4). Evidence indicates that caregivers often operate within fragmented service environments characterized by limited coordination across home care, community services, and long-term care, as well as variable access to information, respite, and system navigation support (5–7). These conditions suggest that caregiver experiences are shaped not only by the demands of providing care, but also by how health systems are organized and how effectively they support integration across sectors (6, 8).

Within this context, caregiver experiences must be understood as both a product and a determinant of health system performance. The ability of health systems to support aging in place, reduce avoidable institutionalization, and ensure continuity of care is closely linked to the capacity and sustainability of unpaid caregiving. However, persistent gaps in system integration and caregiver inclusion in care planning raise important questions about how current models of care distribute responsibility across formal and informal sectors, and with what consequences for system sustainability (5).

The COVID-19 pandemic provides a critical lens through which to examine these dynamics. Rather than representing a discrete disruption, the pandemic intensified and exposed pre-existing structural conditions within health and social care systems (9). Public health restrictions and service disruptions altered care delivery across settings and increased reliance on unpaid caregivers at a time when formal supports were constrained. Canadian evidence documented heightened psychological strain among caregivers during this period (10). However, many of the challenges reported, including difficulties accessing services, reduced system navigation support, and exclusion from decision-making processes, reflected longstanding system design issues that became more visible under conditions of crisis (11). In this sense, the pandemic functioned as a stress test of caregiving systems, revealing the extent to which caregiver wellbeing is contingent on the accessibility, coordination, and responsiveness of health and social care infrastructure (8, 11).

Although caregiving has been widely studied in relation to individual-level outcomes, substantially less attention has been paid to the system-level mechanisms through which health and social care organization shapes caregiving experiences. In particular, there remains limited empirical understanding of how caregivers navigate interactions with integrated and fragmented service systems, and how these interactions influence access to support, management of care demands, and the sustainability of caregiving over time. This gap is consequential given increasing policy emphasis on strengthening home and community-based care, improving system integration, and reducing pressure on institutional services. A better understanding of caregiving as an embedded component of health system organization and performance is therefore needed to inform policies aimed at improving care coordination and supporting the sustainability of health systems that depend on informal care.

Accordingly, this study explores how unpaid caregivers in Ontario, Canada retrospectively experienced and made sense of caregiving during and following the COVID-19 pandemic while supporting older adults living with chronic illness, frailty, or complex conditions. The study focuses on caregivers' interactions with home care, community care, and long-term care systems, with particular attention to how system organization shaped access to emotional, social, practical, and respite supports. By situating caregiving within health system structures and service delivery pathways, this study investigates persistent system-level barriers with implications for more integrated, responsive, and sustainable caregiver-inclusive care across Ontario.

### 1.1 Study aim

The aim of this study was to examine how unpaid caregivers in Ontario experienced and made sense of caregiving during and following the COVID-19 pandemic, and how these experiences were shaped by interactions with health and social care systems. The study addressed the following research questions:

1. How do caregivers describe and interpret their caregiving experiences during and following the COVID-19 pandemic?
2. How do health and social care system structures influence caregivers' access to support and their ability to sustain caregiving?
3. How do interactions across home care, community care, and long-term care systems shape caregivers' experiences over time?

## 2 Methods

### 2.1 Study design

This study employed an interpretive description (ID) approach to explore caregivers' perspectives on existing support services, identify unmet needs, and elicit recommendations for improving service delivery and addressing priority areas of support. ID is well suited to examining complex experiential phenomena in applied health contexts and generating findings that are both analytically rigorous and directly relevant to practice and policy (12). Grounded in a constructivist epistemology, ID enables researchers to move beyond surface-level description to develop interpretive understandings that can inform real-world decision-making and system-level improvements (12).

This study forms part of a broader research project examining caregivers' experiences of supporting older adults. In alignment with its aims, the research questions were intentionally framed to generate actionable insights, extending beyond purely theoretical understanding. ID has been widely used in health and caregiving research to examine lived experiences and translate findings into practical recommendations for service development and policy enhancement (13). This approach therefore provided an appropriate methodological framework for capturing caregivers' perspectives while maintaining a clear focus on applied outcomes.

### 2.2 Sampling and participants

Participants were recruited using purposive and snowball sampling. A maximum variation approach was employed to capture diversity in caregiver characteristics, including relationship to the care recipient (e.g., spouse, adult child, other) and geographic location (Northern and Southwestern Ontario). Eligible participants were caregivers aged ≥18 years who had provided at least 6 months of direct (full- or part-time) care to an older adult (≥60 years) with chronic illness or age-related frailty. Participants were required to reside in Ontario, communicate in English and provide informed consent. Exclusion criteria included paid caregivers, those caring for children or younger adults, individuals residing outside Ontario, and those unable or unwilling to provide consent.

### 2.3 Recruitment and data collection

Multiple recruitment strategies were used to maximize reach and capture a range of caregiving experiences across contexts. Study materials were distributed via email to community-based organizations and professionals supporting caregivers and older adults, who further disseminated information through newsletters, social media, and other communication channels. A recruitment poster, including a study link, was also shared via a research team member's professional social media platforms (LinkedIn and X). Recruitment continued until thematic sufficiency was reached within the study sample, as no new concepts were emerging in the final interviews; however, this reflects sufficiency within the recruited sample rather than saturation across caregiving populations more broadly.

Data were collected between November 2024 and September 2025 through semi-structured interviews lasting approximately 60 min, conducted in person, by telephone, or via Zoom videoconferencing. Participants also completed a socio-demographic questionnaire capturing characteristics such as age, sex, number of care recipients, relationship to the care recipient, and employment status. Interviews were guided by open-ended questions, allowing participants to elaborate on their caregiving experiences in depth. All participants were offered a $25 gift card following their interview in recognition of their time. Interviews were audio-recorded, transcribed verbatim, anonymized, and verified for accuracy prior to analysis.

### 2.4 Thematic analysis

Data were analyzed using Braun and Clarke's (14). six-phase reflexive thematic analysis (Table 1), consistent with ID methodology and its emphasis on generating clinically meaningful, interpretive insights grounded in participants' perspectives (12, 14). Reflexive thematic analysis offers a flexible yet systematic approach to qualitative inquiry and is well suited to inductive analysis across diverse epistemological traditions (15).

**Table 1:** Practical applications of Braun and Clarke's reflexive thematic analysis.

Phases of thematic analysis	Practical application for researchers
1. Familiarization with the data	Researchers immerse themselves in the dataset through repeated reading of raw data (e.g., transcripts and field notes), documenting early reflections and analytic observations through memo writing.
2. Generating initial codes	Data are systematically examined and coded to identify meaningful features relevant to the research question, capturing both explicit (semantic) and underlying (latent) meanings. Coding is flexible, iterative, and comprehensive, and may be conducted manually or using qualitative data analysis software (e.g., NVivo).
3. Searching for themes	Codes are examined and clustered into candidate themes that represent patterned meaning across the dataset. This phase emphasizes active interpretation rather than code aggregation.
4. Reviewing themes	Themes are reviewed for coherence, distinctiveness, and alignment with the dataset and research aims. Themes may be revised, merged, or discarded through recursive engagement with the data.
5. Defining and naming themes	Themes are clearly articulated by defining their core meaning, scope, and relevance. Concise, analytic names are assigned, supported by illustrative data excerpts.
6. Writing reporting	Findings are presented through a coherent analytic narrative that integrates themes, data extracts, and interpretation, situating results within the broader literature and ensuring transparency in analytic decisions.

Analysis began with repeated readings of transcripts by two research team members (SB and KK) to achieve immersion and familiarity with the data. The team independently reviewed transcripts and held reflexive discussions to examine analytic decisions, explore alternative interpretations, and strengthen conceptual clarity. NVivo 15 software supported data management, coding, and theme development. In search for themes, transcripts were systematically double-coded to identify semantic and latent meanings, with discrepancies reconciled through discussion. *In vivo* coding preserved participants' language and perspectives. Codes were iteratively examined and clustered to identify patterns and develop preliminary themes, with ongoing movement between data, codes, and emerging themes to refine interpretations and enhance analytic depth and coherence.

### 2.5 Rigor and reflexivity

Rigor was ensured through in-depth, semi-structured interviews and iterative thematic analysis, with analytic decisions reviewed collaboratively by the research team with expertise in caregiving. Dependability and confirmability were supported by a detailed audit trail documenting methodological decisions, coding, and theme development. Reflexivity was maintained throughout, with the team considering their positionality and potential influence on data collection and interpretation in the sensitive context of caregiving during the pandemic.

Credibility was strengthened through multiple strategies, including triangulation of interviews, independent analysis of transcripts and researcher notes by two team members, and regular team discussions. Transferability was supported through maximum variation sampling, rich descriptions of participants' ages, educational backgrounds, economic and kinship contexts, and caregiving settings. The study was reported in accordance with the Consolidated Criteria for Reporting Qualitative Research (COREQ) and guided by the criteria proposed by Guba and Lincoln.

### 2.6 Ethical considerations

This study is part of a larger study exploring caregivers' experiences of older adults and obtained ethical approval from the Hamilton Integrated Research Ethics Board (HIREB #16443). All participants were provided with a participant information sheet and those who participated provided both oral and written informed consent prior to participation. They were explicitly informed of their right to pause or terminate the interview at any time given the sensitive nature of the topics discussed; however, no participants chose to discontinue or requested additional support. As part of the debrief, participants were provided with information about relevant support organizations. Data collection, storage, and analysis adhered strictly to the Research Ethics Board guidelines to ensure confidentiality, anonymity, and the secure handling of all study materials. All information regarding the participants has been anonymized and participants are identified by a participant number within this manuscript.

## 3 Results

### 3.1 Characteristics of participants

Fifteen caregivers participated in the study. The majority were female (*n* = 14) and primarily adult children of the care recipient (*n* = 9). Caregiver ages ranged from 30 to ≥60 years, eight providing care for 3–5 years and co-resided with the care recipient (*n* = 13). Detailed participant characteristics are presented in Table 2. The semi-structured interviews elicited in-depth perspectives on caregivers' experiences with, and perceptions of, the appropriateness of existing interventions and support resources.

**Table 2:** Sociodemographic characteristics of participants (*n* = 15).

Demographic characteristic	Respondents *n* (%)
Sex	
Female	14 (93.3)
Male	1 (6.7)
Age	
30–39 years	3 (20)
40–49 years	2 (13)
50–59 years	4 (27)
≥60 years	6 (40)
Length of caregiving	
≤ 2 year	2 (13)
3–5 years	8 (53.3)
6–10 years	2 (13)
11–15 years	1 (6.7)
≥15 years	2 (13)
Number of care recipient	
one person	11 (73.3)
two people	2 (13)
≥3 people	2 (13)
Relation to care recipient	
Parent	9 (60)
Spouse	6 (40)
Living with care recipient	
Yes	13 (86.7)
No	2 (13)
Employed	
Yes	9 (60)
No	6 (40)
Employment status	
Full-time	5 (33.3)
Part-time	4 (26.7)
Marital status	
Single	3 (20)
Married	8 (53.3)
Divorce/Separate	2 (13)
Widowed	1 (6.7)
Race (or ethnicity)	
East Asian	2 (13.3)
Black/African descent	1 (6.7)
Hispanic/Latino	2 (13.3)
White/European descent	8 (53.3)
Other	2 (13.3)

### 3.2 Themes

**Figure 1:**
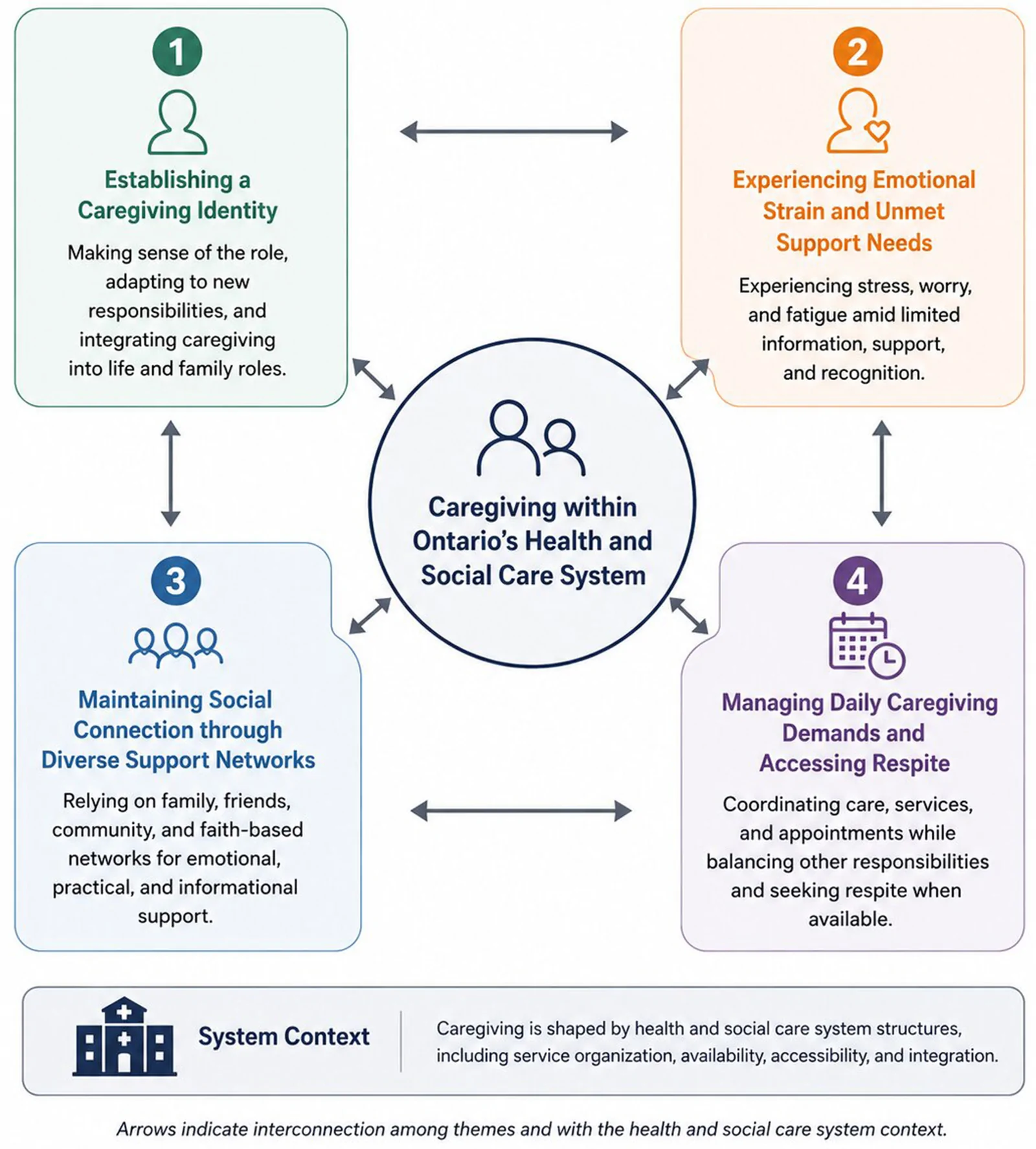
Conceptual framework of unpaid caregivers' experiences within Ontario's health and social care system. The figure illustrates four interconnected themes that describe how unpaid caregivers understood, experienced, and navigated caregiving while supporting older adults living with chronic illness, frailty, or complex conditions: **(1)** establishing a caregiving identity; **(2)** experiencing emotional strain and unmet support needs; **(3)** maintaining social connection through diverse support networks; and **(4)** managing daily caregiving demands and accessing respite. The themes highlight how caregivers' experiences are shaped by service organization, accessibility, coordination, and support structures.

Analysis generated four interconnected themes that captured how caregivers understood, experienced, and navigated caregiving within Ontario's health and social care system: (1) *establishing a caregiving identity*; (2) *experiencing emotional strain and unmet support needs;* (3) *maintaining social connection through diverse support networks*; and (4) *managing daily caregiving demands and accessing respite*. Figure 1 presents a thematic map illustrating the relationships among these findings and their embeddedness within broader care structures and supports. Detailed descriptions of each theme and its associated subthemes, supported by illustrative participant quotations, are presented below.

#### 3.2.1 Theme 1: establishing a caregiving identity

The first theme captures how caregivers constructed a sense of caregiving identity, through recognizing caregiving as a distinct role and navigating broader societal understandings of what it means to be a caregiver. Across participants' accounts, recognition emerged as an important pathway to awareness of available supports and services. Many participants initially understood their role through existing family or relational identities, such as daughter, spouse, or neighbor, rather than identifying themselves as caregivers. As a result, several described overlooking, delaying, or not engaging with caregiver-specific resources, even when information about these supports was available to them (P-04; P-12). One caregiver explained:

“*If people don't even know where to look or don't even recognize themselves as caregivers, they're not going to find these resources. Back then, before COVID, I had no idea where to look. I didn't even recognize myself as a caregiver. And so, even when I would see a pamphlet at the doctor's office, I would say, ‘Oh, no, I'm not a caregiver. I'm just my mom's daughter.” (P-12)*

Across interviews, participants described caregiver recognition as something that often occurred informally and inconsistently rather than through explicit identification processes. Caregivers frequently linked awareness of supports to whether they had encountered information, education, or conversations that helped them view caregiving as a distinct role. As one participant stated:

“*Because you don't see yourself. So, there is this relationship. So there needs to be, first, education about the identity of caregiving.” (P-05)*

Participants also emphasized the broader social visibility of caregiving. Many described caregiving as a common experience that spans the life course and argued that greater public awareness could help normalize caregiving and improve understanding of caregiver needs. One caregiver explained:

“*Is a continuum… from young children all the way up to aging parents… We need to start building societal solutions.” (P-05)*

Clinical and community settings were frequently identified as locations where recognition of caregivers could occur. Participants described interactions with healthcare providers, hospitals, and care transitions as important points at which caregiving responsibilities became visible. One caregiver noted:

“*That's where I think the clinical settings have such huge potential to be able to recognize caregivers, and to be able to connect them to resources, to at least open that door.” (P-12)*

Several participants also highlighted transitions in care, such as hospital discharge, as moments when caregiving responsibilities intensified and information about available supports was particularly needed (P-04). In addition, participants advocated for ongoing caregiving education beginning early in life and continuing across educational settings (P-05; P-09). Collectively, participants' accounts suggest that establishing a caregiving identity involved multiple points of recognition, including self-recognition, public awareness, and interactions within clinical and community settings. Across these accounts, access to information and support was closely connected to whether caregiving was acknowledged as a distinct role.

#### 3.2.2 Theme 2: experiencing emotional strain and unmet support needs

The second theme captures caregivers' experiences of emotional strain and the ways in which their mental health needs remained largely unaddressed within interactions with services and supports. Participants consistently described emotional wellbeing as a central aspect of caregiving and frequently recounted experiences in which their own emotional needs received little attention. One caregiver emphasized:

“*Emotional support is a very important part of my caregiver needs.” (P-10)*

A recurring pattern across interviews was that caregivers reported significant emotional distress while receiving limited direct inquiry, assessment, or support related to their own wellbeing. Several participants described never being asked about their mental health despite prolonged caregiving responsibilities. One caregiver explained:

“*Counseling or therapy was never offered to me. No one asked how I was feeling or if I needed help. I wish there were more caregiver-specific mental health supports.” (P-1)*

Participants frequently described emotional challenges including stress, anxiety, depression, sleep disruption, and exhaustion. Another caregiver reflected:

“*Mental health support would help my physical health. I became very depressed, didn't eat or sleep well, and it took a toll on me.” (P-13)*

Across accounts, caregivers described a mismatch between the emotional demands of caregiving and the supports available to them. When mental health resources were discussed, participants often reported difficulty locating relevant supports or uncertainty about what services were available. Some participants identified community-based supports as a potentially valuable source of assistance. As one caregiver stated:

“*Communities could provide better mental health supports.” (P-12)*

Overall, participants described emotional support as an important but inconsistently available component of caregiving support. Their accounts highlighted recurring experiences of emotional distress alongside limited engagement with services specifically focused on caregiver wellbeing.

#### 3.2.3 Theme 3: maintaining social connection through diverse support networks

The third theme captures how caregivers sought to maintain social connection and reduce isolation through a range of formal and informal support networks. Participants described drawing on multiple sources of support, including peer support groups, individualized support relationships, volunteering, and research participation. Across interviews, caregivers emphasized that no single approach met everyone's needs, with preferences shaped by individual circumstances, geographic location, and comfort with different forms of engagement. Consequently, participants highlighted the importance of having access to diverse opportunities for connection and support.

Group and peer supports were frequently described as valuable opportunities for connection, shared learning, and validation. Participants often emphasized the importance of connecting with others who had similar caregiving experiences. One caregiver expressed a desire for:

“*A young caregiver support group to connect with others going through a similar journey.” (P-01)*

Access to these opportunities, however, varied across participants. Geographic location was frequently discussed as influencing participation. One caregiver explained:

“*Ontario is a big province. If a support group existed in my city, I would attend. Hospice, end-of-life care, and grief support groups are needed locally.” (P-10)*

Participants described group settings as providing opportunities for mutual support and reducing feelings of isolation. One caregiver noted:

“*The advantage of a group is mutual support, communication, and a safe space to vent.” (P-03)*

Similarly, another explained:

“*Group settings help people realize they're not alone, relate to others, offer guidance, build connections with others who are going through a similar situation, and reduce social isolation.” (P-12)*

However, participants also described variation in the perceived usefulness of group-based supports. Some reported that group discussions focused heavily on frustrations without generating practical guidance. One caregiver stated:

“*Not helpful because participants only complained about the system… It would be helpful if moderators offered suggestions or solutions.” (P-11)*

Others preferred individualized forms of support. One participant explained:

“*I don't want to share my story in a group and hear others'. I don't feel comfortable in that sort of context. I prefer individual support, even if it's just a few sessions over months.” (P-02)*

Individualized support was often described as facilitating learning and relationship-building. As one caregiver noted:

“*One-on-one support is where learning happens. I was partnered with a coach in the same situation, which was very helpful.” (P-06)*

Volunteering and research participation also emerged as meaningful sources of connection. One caregiver stated that:

“*Volunteering helps with the social aspect and provides invigorating engagement.” (P-09)*

Similarly, participants described research involvement as creating opportunities to connect with other caregivers. One participant reflected:

“*Even just participating in a meeting with other caregivers [as part of participating in a focus group discussion for a research study] was helpful. I was able to build small connections, understand others' points of view, and gather insight on what's helpful in caregiving.” (P-10)*

Another noted:

“*Even being involved in research can connect you with peer support.” (P-05)*

Collectively, participants' accounts highlighted the diversity of social support experiences available to caregivers. They described differing preferences for group, individualized, and informal forms of connection, while also noting variation in availability, accessibility, and perceived usefulness.

#### 3.2.4 Theme 4: managing daily caregiving demands and accessing respite

The fourth theme captures how caregivers managed the practical demands of day-to-day caregiving while seeking opportunities for respite and relief. Across interviews, participants consistently described respite as an important resource for sustaining their caregiving role, yet one that was often difficult to access. Many caregivers reported that limited respite options affected their ability to attend programs, participate in activities, or take breaks from caregiving responsibilities. One caregiver explained:

“*Some people can't attend programs because they can't find care for their loved ones. There are few respite services for adults or the elderly.” (P-10)*

Similarly, another participant noted:

“*Many caregivers struggle to find respite services to leave the house for a short break without worrying about their loved one.” (P-01)*

Participants described respite as a necessity rather than an optional support. One caregiver stated:

“*Access to free and easily available respite is crucial… Even short visits from a PSW [personal support worker] once or twice a week don't always provide enough relief.” (P-12)*

Cost was also frequently discussed when caregivers described respite services. One participant explained:

“*Homes for the aged offer respite care, but it's expensive—around $1,700 for a week. That's not cheap.” (P-15)*

In addition to respite, caregivers described challenges related to household tasks, personal care, and physically demanding caregiving activities. Several participants reported requiring assistance with tasks that exceeded their physical capacity. One caregiver stated:

“*I physically can't do some tasks, like getting him to wound care. I need help with the physical aspects of caregiving and day-to-day duties.” (P-11)*

Another participant described challenges with

“*Giving the person I care for a shower or helping them transfer from bed to wheelchair with the lift.” (P-01)*

For some caregivers, these responsibilities contributed to injury and physical strain. One participant explained:

“*For grocery shopping, I needed someone to sit with my husband for 2 h. He's 200 pounds, I'm 150. Lifting, turning, bathing, and cleaning him caused injuries. There's a lot of physical care needed with no support at home.” (P-13)*

Transportation was another commonly reported area of need. One caregiver stated:

“*You can't always get transportation. Drivers are helpful because I can't lift a walker into the car.” (P-15)*

Another noted:

“*Community home care doesn't help with transportation, housekeeping, or food preparation. I have to drive him everywhere.” (P-02)*

Across participants' accounts, respite, transportation, physical assistance, and household support were described as interconnected aspects of managing daily caregiving responsibilities. Caregivers frequently discussed challenges obtaining one or more of these supports while continuing to meet the ongoing needs of the person they cared for.

## 4 Discussion

This study examines how unpaid caregivers in Ontario retrospectively understood and navigated caregiving during and following the COVID-19 pandemic, while exploring how these experiences were shaped through interactions with health and social care systems. Although participants reflected on caregiving during the pandemic period, their accounts pointed to challenges that extended beyond COVID-19 itself. Rather than describing entirely new forms of burden, participants consistently identified patterns in how caregiving was recognized, supported, and sustained across home care, community care, and long-term care contexts. In this sense, the pandemic provided a context in which existing features of caregiving systems became more visible and, in many cases, more consequential. These findings are consistent with evidence suggesting that COVID-19 exposed longstanding vulnerabilities in caregiver support systems rather than creating wholly novel challenges (10, 11).

Across all four themes, a common pattern emerged: caregivers frequently occupied a central role in sustaining care yet remained peripheral within the systems responsible for supporting that care. Participants described difficulties becoming recognized as caregivers, limited attention to their own wellbeing within service encounters, inconsistent access to psychosocial supports, and challenges obtaining respite and practical assistance. Taken together, these findings suggest that caregiver strain was shaped not only by caregiving responsibilities themselves but also by the ways caregivers were positioned within health and social care systems.

The process of establishing a caregiving identity illustrates this dynamic. Participants frequently described understanding themselves primarily as spouses, adult children, family members, or neighbors rather than as caregivers. Recognition of caregiving often occurred only after prolonged involvement in care or through independent discovery of caregiver-related resources. Importantly, participants' accounts suggested that access to support was closely linked to whether caregiving was recognized and named within interactions with services. Clinical encounters, hospital discharges, and other points of contact were frequently described as missed opportunities for caregiver recognition and connection to available resources. These findings align with caregiver identity theory, which conceptualizes caregiving identity as a gradual and negotiated process rather than an immediately assumed role (16, 17). However, the present findings extend this literature by demonstrating that identity formation was not solely an individual process but was also shaped through interactions with health and social care systems that variably acknowledged—or overlooked—the caregiver role.

The findings related to emotional wellbeing similarly point to the significance of caregivers' positioning within care systems. Participants consistently described substantial emotional strain, including stress, anxiety, depression, sleep disruption, and emotional exhaustion. Yet they also reported that discussions about their own wellbeing were largely absent from service interactions. Caregiver distress was often described as something managed privately rather than assessed or addressed through formal support pathways. These findings are consistent with previous research documenting elevated psychological burden among caregivers and limited access to caregiver-specific mental health supports (5, 7). They also extend the stress process model by illustrating how emotional strain accumulated not only through caregiving demands but through repeated encounters in which caregivers' own needs remained secondary to those of care recipients (18).

Social connection emerged as another important dimension through which caregivers navigated support. Participants described peer groups, mentoring relationships, volunteering, and research participation as valuable opportunities for connection, validation, and shared learning. At the same time, access to these supports appeared uneven and dependent on individual circumstances, geographic location, and personal preferences. The variability observed across participants' accounts suggests that opportunities for social connection were not experienced as a routine component of caregiving support but rather as resources that caregivers encountered inconsistently. These findings support previous evidence demonstrating the protective effects of social support on caregiver wellbeing and resilience (4, 19). They also highlight the importance of offering diverse forms of support that accommodate differing preferences for group-based and individualized engagement.

The findings regarding respite and practical support further illustrate how caregiving experiences were shaped through interactions with service systems. Participants consistently described respite as essential to sustaining caregiving over time, yet frequently encountered barriers related to availability, affordability, timing, and eligibility. Similar patterns were evident in relation to transportation assistance, household support, personal care, and physically demanding caregiving tasks. Rather than being discussed as isolated needs, these supports were often described as interconnected components of everyday caregiving. When unavailable or difficult to access, caregivers reported absorbing these responsibilities themselves, often at considerable physical and emotional cost (11, 20). These findings are consistent with literature identifying respite and practical assistance as critical determinants of caregiver wellbeing (19, 21, 22). More broadly, they suggest that caregiver strain is closely connected to the extent to which essential supports are available, accessible, and responsive to the realities of day-to-day caregiving.

Taken together, the findings demonstrate that caregiver experiences are shaped through recurring patterns of interaction with health and social care systems. Across themes, participants described challenges related not only to caregiving demands but also to how caregivers were identified, engaged, and supported within service environments. The findings therefore shift attention from caregiving as an individual experience of burden toward caregiving as a role that is mediated by organizational arrangements, service pathways, and access to support. Recognizing these broader influences is important for understanding how caregiver strain emerges and why unmet needs persist despite the essential contribution of unpaid caregivers to Ontario's care system (3, 5, 6).

### 4.1 Limitations and future directions

This study has some methodological and contextual limitations that should be considered when interpreting the findings. The study was conducted within a single Canadian province, and the organization of health and social care services in Ontario may differ from other jurisdictions, potentially limiting transferability of the findings. Although the predominance of female participants reflects well-established gendered patterns of caregiving, the study was based on a relatively small qualitative sample consisting primarily of female, adult-child, co-residing, and English-speaking caregivers. Consequently, the findings may not fully reflect the diversity of caregiver experiences across Ontario, as support needs and interactions with health and social care systems may vary across gender, caregiving relationships, living arrangements, and linguistic or cultural contexts. While one-time interviews provided rich retrospective accounts, they did not capture how caregiving experiences and support needs evolved over time. Finally, some interviews were conducted by telephone, limiting opportunities to observe non-verbal forms of communication that may have further enriched data interpretation.

Future research should employ longitudinal designs to examine how caregiving experiences and interactions with health and social care systems evolve over time, including how caregiver strain develops across different stages of the caregiving trajectory. Comparative studies across provinces and health systems would further clarify how policy environments and service structures shape access to supports and caregiver outcomes. Additional work is needed to better capture the experiences of underrepresented caregiver groups, particularly male caregivers and those from diverse cultural, linguistic, socioeconomic, and geographic backgrounds. This would strengthen understanding of variation in how caregivers engage with and navigate health and social care systems, as well as potential inequities in access to supports. Future studies should also use mixed-methods and implementation-focused approaches to evaluate strategies aimed at improving caregiver identification, assessment, service coordination, respite access, and navigation across care sectors. Continued attention to caregiver mental health and social wellbeing remains essential for understanding how caregiving is shaped by both individual circumstances and broader system-level arrangements.

### 4.2 Implications for policy and practice

The findings indicate that supporting caregivers requires more than expanding individual services; it requires addressing the organizational arrangements through which caregivers are identified, engaged, and supported across home care, community care, and long-term care systems. Across participants' accounts, unmet needs were linked to recurring system-level gaps, including inconsistent caregiver identification, limited attention to caregiver wellbeing, fragmented access to services, and insufficient respite and practical supports. Addressing these challenges requires embedding caregiver support within routine care pathways rather than relying on self-identification, self-navigation, or crisis-driven access to services.

A foundational intervention is the implementation of standardized caregiver identification processes across primary care, hospital discharge planning, Ontario Health atHome intake, and long-term care admission procedures. Many participants entered care systems through relational roles such as spouse, daughter, or family member without being connected to available supports. Embedding caregiver identification within intake workflows and electronic health records would facilitate earlier access to information, referral pathways, and support services, aligning with national efforts to formalize caregivers as essential care partners within health systems (23–25). Effective implementation should extend beyond documentation to include structured opportunities for caregiver involvement in care planning, communication, and decision-making where appropriate. Healthcare organizations can support this process through caregiver-inclusive tools and practices, such as the Essential Care Partner Support Hub, that integrate caregivers more consistently into care teams and care processes.

Recognition alone is insufficient without systematic attention to caregiver wellbeing. Participants frequently described substantial emotional strain while reporting that their own wellbeing was rarely addressed within service interactions. Routine caregiver strain screening integrated into primary care visits, hospital discharge processes, and home care reassessments could facilitate earlier identification of distress and more timely referral to mental health, respite, and community-based supports. Social prescribing may provide a complementary mechanism for connecting caregivers to peer support, counseling, culturally responsive programs, and other community resources that reduce isolation and strengthen emotional wellbeing (22).

The findings also point to the need for stronger care coordination. Caregivers often described independently navigating multiple disconnected services while arranging respite, transportation, and practical assistance. Establishing dedicated caregiver navigation roles within Ontario Health Teams and home and community care systems could improve continuity of care, reduce administrative burden, and enhance access to available supports. Integrated navigation models have shown particular promise in complex care systems where fragmentation creates barriers to timely service access (22, 25).

Persistent gaps in respite and practical support point to the need for expanded, accessible, and flexible service infrastructure. Participants consistently described respite as essential to sustaining caregiving, yet difficult to access due to barriers related to availability, cost, eligibility, and flexibility. Expanding subsidized respite options—including in-home, short-stay, and emergency respite services—would address a critical and recurring unmet need. In parallel, home support programs should be expanded to include transportation, household assistance, and personal care, reflecting the full scope of responsibilities assumed by unpaid caregivers. Evidence consistently demonstrates that respite and practical supports reduce caregiver strain and support caregiver wellbeing (19, 22).

Although financial concerns were not always discussed explicitly, they were evident through participants' descriptions of service costs, reduced employment participation, and reliance on personal resources. Strengthening financial protections through expanded caregiver tax credits, enhanced Employment Insurance Caregiver Benefits, and targeted supports for high-intensity caregiving situations may help mitigate the economic consequences of sustained caregiving. Existing programs remain limited in accessibility and reach, leaving many caregivers without adequate financial protection over prolonged periods of care (23, 26).

Collectively, the findings support the development of an integrated caregiver pathway across Ontario's health and social care system. Such a pathway would connect caregiver identification, assessment, navigation, respite, psychosocial resources, and financial assistance within a coordinated continuum of care rather than a collection of disconnected programs. Embedding these components within routine care processes could improve equity, continuity, and accessibility while reducing the responsibility currently placed on caregivers to independently locate, navigate, and coordinate services (6, 25). Existing caregiver organizations and community-based programs provide a strong foundation for this approach and could be more systematically integrated into formal care pathways (23, 24). Advancing a coordinated caregiver pathway represents an important opportunity to strengthen caregiver wellbeing while enhancing the responsiveness and long-term sustainability of Ontario's health and social care system.

## 5 Conclusion

Across home care, community care, and long-term care settings in Ontario, unpaid caregiving is shaped not only by the demands of providing care but also by how caregivers are recognized, assessed, and supported within health and social care systems. The findings demonstrate that caregiver experiences are shaped through recurring institutional patterns, including the absence of systematic caregiver identification, limited integration of caregiver wellbeing into routine assessment processes, fragmented access to psychosocial and practical supports, and constrained availability of respite and daily assistance services. These patterns were evident across caregivers' accounts of identity formation, emotional strain, social connection, and unmet practical support needs.

Rather than reflecting isolated service gaps, the findings suggest that caregiver strain is embedded within the routine organization and delivery of care. Although participants reflected on experiences during and following the COVID-19 pandemic, their accounts point to longer-standing structural conditions that influence caregiving across care settings. By centering caregivers' perspectives, this study demonstrates how system-level arrangements shape the conditions under which unpaid care is sustained and reframes caregiver strain as a consequence of how care systems are organized rather than solely an outcome of individual caregiving responsibilities. Recognizing and responding to these structural conditions is essential if health and social care systems are to support caregivers effectively and sustain the unpaid care on which they increasingly depend.

## Data Availability

The raw data supporting the conclusions of this article will be made available by the authors, without undue reservation.
