# Correction: Caring amid crisis: a qualitative study of caregivers' experiences, wellbeing, and support for older adults

**DOI:** 10.3389/fpubh.2026.1989158

**Published:** 2026-09-16

**Authors:** Sheila A. Boamah, Kyla J. Kovalik

**Affiliations:** 1 School of Nursing, Faculty of Health Sciences, McMaster University, Hamilton, ON, Canada; 2 Department of Health, Aging & Society, Faculty of Social Sciences, McMaster University, Hamilton, ON, Canada

**Keywords:** care coordination, caregiver experiences, health and social care systems, older adults, Ontario, quality of life, unpaid caregivers

In the published article, the **Acknowledgments** section was missing. The correct **Acknowledgments** appear below:

“The authors gratefully acknowledge the caregivers (carers) who generously shared their time and experiences, which were essential to this study. We also thank Hoda Herati for their contributions to data collection and initial coding.”

The original version of this article has been updated.

